# Study of the Compressive Properties of Heavy Calcium Carbonate-Reinforced Epoxy Composite Spheres (HC-R-EMS) Composite Lightweight Concrete

**DOI:** 10.3390/polym15051278

**Published:** 2023-03-02

**Authors:** Rong Ma, Zheng Cao, Tao Jiang, Ying Wang, Shanshan Shi, Wenge Li, Yuantao Zhao, Ning Zhong, Danda Shi, Xinfeng Wu

**Affiliations:** 1 Merchant Marine College, Shanghai Maritime University, Shanghai 201306, China; 2 College of Ocean Science and Engineering, Shanghai Maritime University, Shanghai 201306, China; 3 School of Energy and Materials, Shanghai Polytechnic University, Shanghai 201209, China

**Keywords:** heavy calcium carbonate, lightweight concrete, low density, compressive strength, hollow glass microspheres, basalt fiber

## Abstract

Lightweight concrete is one of the effective means to solve the problems of structural component weight, energy efficiency, and fire safety in modern civil engineering. Heavy calcium carbonate-reinforced epoxy composite spheres (HC-R-EMS) were prepared by the ball milling method, and HC-R-EMS, cement, and hollow glass microspheres (HGMS) were mixed into the mold by the molding method to prepare composite lightweight concrete. The relationship between the HC-R-EMS volumetric fraction, the initial inner diameter of the HC-R-EMS, the number of layers of HC-R-EMS, the HGMS volume ratio, the basalt fiber length and content, and the multi-phase composite lightweight concrete density and compressive strength was studied. The experimental results show that the density of the lightweight concrete ranges between 0.953–1.679 g/cm^3^ and the compressive strength ranges between 1.59–17.26 MPa, where the volume fraction of HC-R-EMS is 90%, the initial internal diameter is 8–9 mm, and the number of layers of HC-R-EMS is three. The lightweight concrete can meet the requirements of high strength (12.67 MPa) and low density (0.953 g/cm^3^). In addition, the addition of basalt fiber (BF) can effectively improve the compressive strength of the material without changing the density of the material. From a micro-level perspective, HC-R-EMS is closely combined with the cement matrix, which is conducive to increasing the compressive strength of concrete. Basalt fibers connect the matrix into a network, improving the maximum limit force of the concrete.

## 1. Introduction

Modern civil engineering [1] continuously puts forward new requirements for the weight, energy efficiency, and fire safety of structural components. The application of lightweight concrete [2,3,4] can serve as a solution to these demands. As a modern composite material, lightweight concrete is not only considered a building material, but also a thermal insulation material [5,6] or aesthetic architectural cladding [7]. The basic characteristics of lightweight concrete [8] are a lower bulk density and an improved thermal insulation performance, which is an important difference from ordinary concrete. Additionally, the lightweight aggregate in lightweight concrete is usually an expanding material [9], which is highly effective for soundproofing [10,11] and insulation [12,13]. In terms of seismic resistance [14,15], lightweight aggregate concrete has the inherent advantage of being lightweight, and using it to partially or completely replace normal weight concrete can lead to significant benefits by reducing the structural dead load.

Lightweight concrete is used in the construction industry [16] to make precast concrete slabs, blocks, and panels, and these materials are widely used in the building industry because of their low weight and ease of transportation and handling. Due to its low density and thermal conductivity, lightweight concrete is also used as an insulating material [17], and it is commonly used in roofs, walls, and floors to provide insulation and reduce heat loss. Lightweight concrete can also be used for fire protection purposes [18] because of its good fire resistance properties. It is often used in high-rise buildings, tunnels, and bridges to provide a protective layer against fire. Lightweight concrete is also used for soundproofing purposes [19] because of its good acoustic properties. In addition, lightweight concrete has been explored as a marine floating and deep-sea material [20].

Lightweight concrete can be made by introducing foaming agents into the concrete mixture [21] or by filling the concrete with lightweight aggregates [2,22]. This can effectively reduce the density of the concrete. Different types of aggregates and controlled replacement ratios [23] can be used to improve the performance of the concrete based on the desired properties. Foamed concrete [24], also known as air concrete, is made by introducing a foaming agent, which is lightweight and has low density, good thermal insulation performance, good sound insulation and noise reduction performance, excellent seismic performance, etc. However, its compressive strength is relatively low. Filled lightweight concrete includes EPS concrete [25,26,27], HGMS concrete [28,29,30], ceramic ball concrete [31,32,33], plastic concrete [34], etc.

Hollow glass microspheres (HGMS) [30] have developed into a new type of high-performance lightweight material, with the characteristics of reducing costs, reducing weight, and ease of processing and construction. They are commonly used as fillers in composite materials. HGMS’ particle size is 2–150 microns and their density range is 0.15–0.6 g/cm^3^. Using HGMS is environmentally friendly because they contain 45% recycled glass. The spherical shape and smooth surface of HGMS can be used to change the flowability of cement mortar and adding HGMS in lightweight concrete can promote fiber dispersion. Due to its low density, high compressive strength, and good dispersion and flowability, HGMS [24] are widely used in many lightweight concrete materials with high demands.

Expanded polystyrene (EPS) bead is a hard closed-cell foam plastic made by adding a foaming agent to polystyrene resin and heating it to soften, generating gas. Its density is less than 30 kg/m^3^. Early research on EPS [35,36] concrete involved adding millimeter-sized EPS beads to mortar [37,38] or cement to reduce the density and thermal conductivity. Compared to conventional lightweight aggregate concrete, EPS concrete has better workability and volume stability. EPS [39] concrete can be constructed without special machinery, and it is suitable for places where large machinery is difficult to use. Structural elements made of EPS concrete can be manufactured on the construction site and are suitable for emergency rescue, which greatly facilitates the construction process.

In this experiment, HC-R-EMS was prepared by the ball rolling method [40,41,42], using EPS as a light aggregate, wrapping EPS with epoxy resin, and making reinforced epoxy resin balls with heavy calcium carbonate. A HGMS-cement matrix system is prepared by mixing HGMS and cement. The enhanced epoxy resin balls are added to the HGMS-cement matrix as lightweight concrete aggregate to prepare lightweight concrete samples. This paper compares the compressive strength and density of lightweight concrete prepared under different conditions from the stacking volume fraction, inner diameter, enhancement layer number, HGMS-cement matrix HGMS and cement ratio, and the length and content of basalt fibers added to the HGMS-cement matrix. The reason for the increase in the compressive strength of lightweight concrete is explored through macro- and micro-graphs [43].

## 2. Materials and Methods

### 2.1. Materials

Epoxy resin (Araldite^®^ LY 1564) and amine curing agent (Aradur ^®^ 3486) were purchased from Huntsman Chemical Co., Ltd., Shanghai, China. The 42.5 Portland cement was purchased from Shenzhen Zhongning Technology Co., Ltd., Shenzhen, China. The Polystyrene foam balls (EPS) were purchased from Hangzhou Hangchao Packaging Materials Co., Ltd., Hangzhou, China. The K1 hollow glass bead (HGMS) was purchased from the 3M Company in Shanghai, China. The compressive strength of K1 is 1.72 MPa, the vacuum density is 0.125 g/cm^3^, and the particle size is 20–80 μm.

### 2.2. Preparation Process of HC-R-EMS

**Figure 1:**
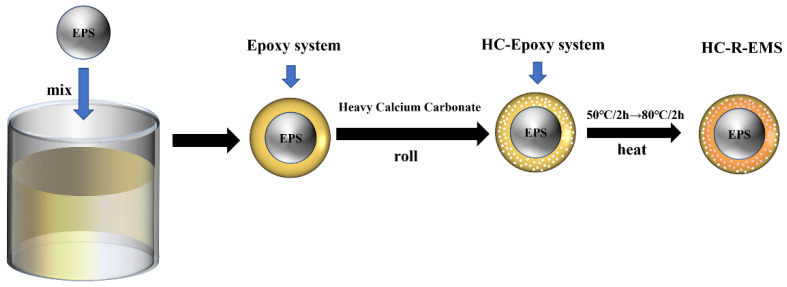
The process of preparing a single layer of epoxy composite ball.

This experiment mixes epoxy resin and a hardener in a 3:1 ratio and uses a deaeration mixer to mix the two at 2000 RPM to form an epoxy curing system. EPS is added to the mixture in a 10:1 ratio of epoxy curing system to EPS, and the mixture is further mixed until the EPS surface is uniformly coated. The EPS balls coated with the epoxy curing system are then mixed with heavy calcium carbonate powder in a rolling ball machine. When the epoxy curing agent-coated EPS ball surface is completely covered with reinforcing material, sieve the excess powder on its surface and pour the EPS balls into the tray, put the tray into a vacuum drying oven at 50 degrees Celsius for 2 h; then, set the vacuum drying oven temperature to 80 degrees Celsius for another 1 h, dry and take out the enhanced epoxy composite ball at room temperature for 24 h after the epoxy curing system is completely cured to make a single layer of material reinforced epoxy composite ball. Figure 1 shows the process of preparing a single layer of epoxy composite ball. By following the same steps and increasing the amount of epoxy curing system used, two-layer and three-layer material-enhanced epoxy composite balls can be made.

### 2.3. Preparation Process of Lightweight Concrete

**Figure 2:**
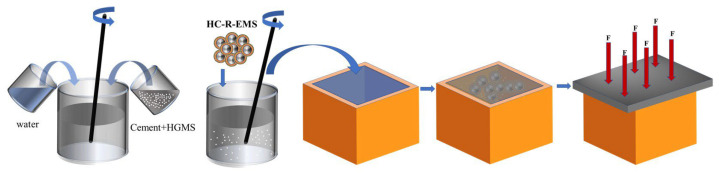
Schematic diagram of the preparation process of lightweight concrete.

This experiment uses GB/T16491-2008 of China as the standard. The maximum test force can be 300 KN and the maximum test range can be 1100 mm. The process for preparing HC-R-EMS lightweight concrete is as follows: a 70.7 mm × 70.7 mm × 70.7 mm mold is selected as the specimen carrier (Figure 2), enhanced epoxy composite balls are poured into the mold to fill it completely, with the mass of the added enhanced epoxy composite balls being 100% of the stacked volume fraction of the enhanced epoxy composite balls. According to different control groups, the corresponding volume fraction of enhanced epoxy composite balls is taken. A suitable amount of 42.5 Portland cement is taken, and HGMS is added and mixed in a 2:3 cement volume ratio, and water is added for further mixing and stirring (Figure 2), creating the HGMS-cement system matrix. The required volume fraction of the enhanced epoxy composite balls and HGMS-cement matrix are poured into a deaeration mixer for stirring, and then filled into the mold together (Figure 2). In order to make the specimen surface smooth and easy to demold, the inside of the mold is completely wiped with gasoline before filling the mold. After the mold is filled, a polyester film is placed on top of the mold to squeeze out the bubbles and a load is applied on top of the mold to solidify the specimen and maintain a smooth surface (Figure 2). After the specimen is cured, it is demolded, and the lightweight concrete block is then cured for 28 days to create a multi-phase composite lightweight concrete. The filling process should be kept tight, and the pore generation should be kept to a minimum, followed by pressing, keeping the mold in a compressed state; the compression time should preferably be kept above 24 h. The sample can be removed from the mold after 48 h of pressing and needs to be placed in a maintenance box at 20 °C and 95% air humidity for 28 days.

### 2.4. Characterization of Composites

Density measurement: Using a digital analytical balance (provided by Guangzhou BGD laboratory instrument supply company), the mass of the sample is measured. The length, width, and height parameters of the sample are measured using a digital caliper, and the volume is calculated. The average density of the sample is calculated using Formula (1).

(1) $${Ρ}_{\mathrm{CH}-R-\mathrm{EMS}}=\frac{\sum_{n=1}^{n=50}{\left(m_{n}\right)}_{HC-R-EMS}}{\sum_{n=1}^{n=50}{\left(V_{n}\right)}_{HC-R-EMS}}$$

Compressive strength test: Use the universal testing machine (CMT5350, Shenzhen San Shi Cross-Technology Co., Ltd., Shenzhen, China) to test the compressive strength of HC-R-EMS lightweight concrete. This experiment is based on the Chinese standard GB/T16491-2008. The maximum test force can reach 300 kN and the maximum test range can reach 1100 mm.

Surface morphology observation: To analyze the bonding issues at the surface of the composite materials, a scanning electron microscope (SEM) (JEM-4701, JEOL, Tokyo, Japan) is used to observe the surface morphology of the material. The sample is cut open to obtain an observation cross-sectional part, and an ultrasonic cleaner (SK2200H, Shanghai Kudos Ultrasonic Instrument Co., Ltd., Shanghai, China) is used to clean the surface dust of the material. Then, a sputtering coater (ISC150) is used to sputter a layer of gold on the surface of the sample in order to more clearly observe the morphological characteristics.

This experiment focuses on the effect of different influencing factors on the performance of lightweight concrete. The influencing factors can be divided into the stacking volume fraction, diameter, and number of layers of the HC-R-EMS; the type of HGMS, the BF content, and length in the matrix. The specific formulations used for the experiments are shown in Table 1.

## 3. Results and Discussion

### 3.1. HC-R-EMS Macroscopic Morphology and Density

**Figure 3:**
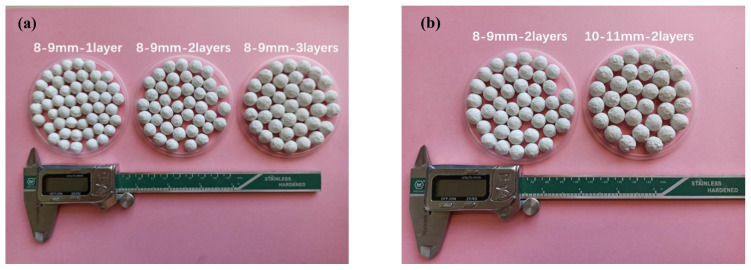

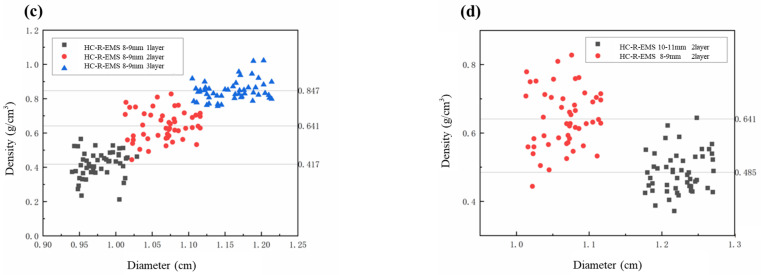
Characterization of HC-R-EMS. (**a**) Photographs of HC-R-EMS with different number of layers. (**b**) Photographs of HC-R-EMS with different diameters. (**c**) Densities of HC-R-EMS with different number of layers. (**d**) Density of HC-R-EMS with different diameters.

Figure 3 shows the macroscopic morphology and density characterization of the HC-R-EMS. From Figure 3a,b, it can be observed that the surface of the HC-R-EMS is complete and without defects, which will help to increase the mechanical strength of the hollow epoxy microsphere and enhance the bonding performance of the epoxy composite sphere with the HGMS-cement matrix and reduce the internal voids in the lightweight concrete.

To avoid the occurrence of unexpected data and to reduce errors as much as possible, 50 HC-R-EMS samples were randomly selected for each group and the diameter and mass of the samples were statistically analyzed. The results show that the more layers of HC-R-EMS on the EPS, the higher the density of the prepared cement/epoxy composite material; the larger the EPS diameter, the lower the density of the prepared HC-R-EMS.

The macroscopic diameter of the HC-R-EMS increases, the thickness of the macroscopic layer increases, and the density of the macroscopic layer increases. When the initial diameter of the EPS small balls is within the range of 8–9 mm, the diameter distribution of the HC-R-EMS is between 9–12 mm. The density of the HC-R-EMS is mainly distributed in three areas: (a) the HC-R-EMS-1 layer, 0.212–0.565 g/cm^3^ (diameter 9.43–10.29 mm); (b) the HC-R-EMS-2 layer 0.444–0.828 g/cm^3^ (diameter 10.13–11.16 mm); (c) the HC-R-EMS-3 layer 0.757–1.023 g/cm^3^ (diameter 11.05–12.14 mm), as shown in Figure 3c.

When the number of layers is the same (two layers), the densities of the prepared HC-R-EMS are 0.641 g/cm^3^ (8–9 mm) and 0.485 g/cm^3^ (10–11 mm). The larger the EPS, the larger the diameter and the lower the density of the prepared HC-R-EMS. As the diameter of the EPS increases, the density of the prepared HC-R-EMS decreases, as shown in Figure 3d.

### 3.2. Characterization of Density and Compressive Strength of Lightweight Concrete Filled with Different Stacking Volume Fractions

**Figure 4:**
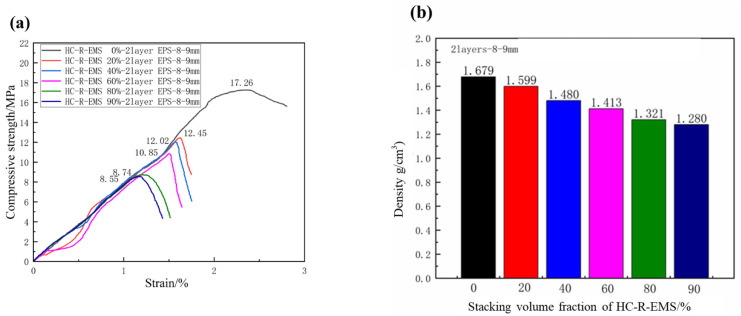
Compressive strength (**a**) and density (**b**) of lightweight concrete filled with different stacking volume fractions of HC-R-EMS.

Figure 4 shows the density and compressive strength of the concrete composite materials under different HC-R-EMS stacking volume fraction conditions. In Figure 4a, the compressive strengths of the concrete composite materials are 17.26 MPa (0%), 12.45 MPa (20%), 12.02 MPa (40%), 10.85 MPa (60%), 8.74 MPa (80%), and 8.55 MPa (90%), respectively. In Figure 4b, the densities of the concrete composite materials are 1.679 g/cm^3^ (0%), 1.599 g/cm^3^ (20%), 1.480 g/cm^3^ (40%), 1.413 g/cm^3^ (60%), 1.321 g/cm^3^ (80%), and 1.280 g/cm^3^ (90%). Under different HC-R-EMS stacking ratio conditions, the compressive strengths of the composite materials are 72.1% (20%) of the pure HGMS-cement matrix strength; 69.6% (40%); 62.9% (60%); 50.6% (80%); 49.5% (90%).

The density of the two-layer HC-R-EMS used in this experiment is 0.641 g/cm^3^, which is far lower than the density of the HGMS-cement-based system (1.679 g/cm^3^). As a result, the density of the concrete composite material decreases as the stacking ratio of the HC-R-EMS increases. When the stacking ratio of the HC-R-EMS is 90%, the density of the concrete composite material is only 1.280 g/cm^3^. Although the strength of the composite material is weakened to some extent, the minimum density of the concrete composite material (1.280 g/cm^3^) can minimize the weight of the material and achieve a lightweight effect.

The compressive strength of the concrete composite material is weakened as the stacking ratio of the HC-R-EMS increases. The main reason for this phenomenon is that the stacking volume fraction of the HC-R-EMS determines the number of holes in the concrete. As the filling amount of the balls increases, the possibility of the HC-R-EMS contacting each other in the HGMS-cement matrix increases. When the material is loaded, the contact points of the balls are easily damaged. In addition, the increase in the amount of HC-R-EMS filler makes it easy for the concrete composites to produce air bubbles in the preparation process and increases the interface defects of the materials, which obviously weakens the compressive strength of the concrete composites.

### 3.3. Characterization of Density and Compressive Strength of Lightweight Concrete Filled with HC-R-EMS of Different Diameters

**Figure 5:**
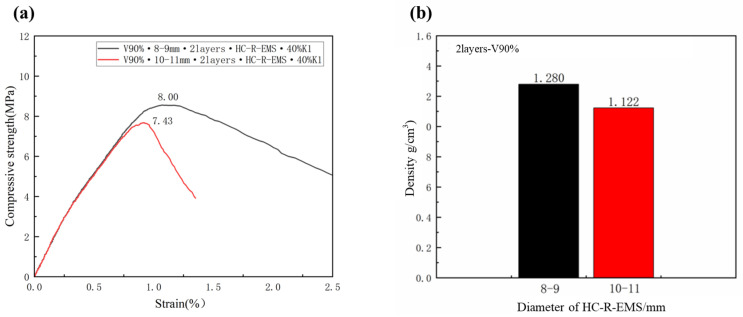
Compressive strength (**a**) and density (**b**) of lightweight concrete filled with different HC-R--EMS diameters.

Figure 5 shows the characterization of the density, compressive strength, and modulus of elasticity of the concrete filled with HE-R-EMS of different inner diameters. From Figure 5a, it can be seen that the compressive strength of the multi-phase lightweight concrete prepared by HC-R-EMS with inner diameters of 8–9 mm and 10–11 mm is 8.55 MPa and 7.68 MPa, respectively. With the increase in the inner diameter, the compressive performance of the HC-R-EMS is weakened; the latter is less than the former. This is because when the inner diameter of the EPS is small, under the same volume fraction of the HC-R-EMS conditions in the same area, the smaller the inner diameter of the HC-R-EMS, the more it is filled, the average pressure each HC-R-EMS bears is small, the deformation of the HC-R-EMS is small, and the compression strength is high. The smaller the diameter of the epoxy composite ball, the faster the force is transmitted on its surface. Figure 5b shows the density of the multi-phase lightweight concrete made from HC-R-EMS with different inner diameters, which are 1.280 g/cm^3^ (8–9 mm) and 1.122 g/cm^3^ (10–11 mm), respectively. The density of the lightweight concrete decreases with the increase in the HC-R-EMS inner diameter; this is because as the initial diameter of the EPS balls increases, the density of the HC-R-EMS decreases with its initial diameter. Thus, the density of the lightweight concrete made from HC-R-EMS with a larger inner diameter also decreases.

### 3.4. The Effect of HC-R-EMS Wall Thickness on the Compressive Strength and Density of Multi-Phase Composite Lightweight Concrete

**Figure 6:**
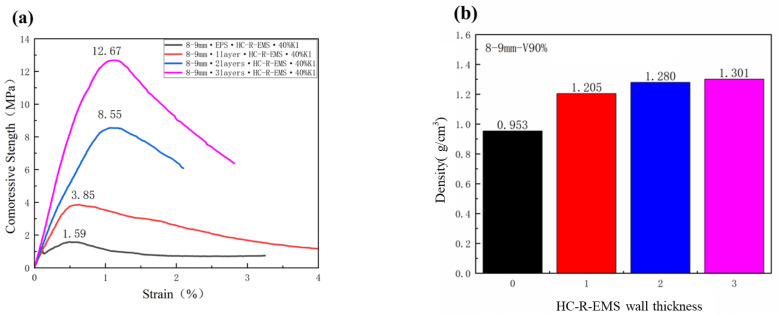
Compressive strength (**a**) and density (**b**) of lightweight concrete filled with different HC-R-EMS wall thickness.

In order to study the effect of the reinforcement layers on the compressive strength of the multi-phase lightweight concrete, the present group of experiments used HC-R-EMS with an inner diameter of 8–9 mm, and the volume fraction of the HC-R-EMS added in the matrix was 90%. One, two, and three layers of reinforcement layers were selected, and the EPS control group was set. As shown in Figure 6, the compressive strengths and densities of the concrete composite materials under different HC-R-EMS wall thicknesses are 1.59 MPa (zero layers), 3.85 MPa (one layer), 8.55 MPa (two layers), and 12.67 MPa (three layers); 0.953 g/cm^3^ (one layer), 1.205 g/cm^3^ (one layer), 1.280 g/cm^3^ (two layers), and 1.301 g/cm^3^ (three layers). From Figure 6a, the compressive strength increases, because as the number of heavy calcium carbonate-reinforced epoxy resin-curing layers outside the EPS increases, the structure of the epoxy composite ball becomes more stable and is less likely to be damaged under stress. In addition, the compressive strength of the multi-phase lightweight concrete increases with the increase in the wall thickness of the filled HC-R-EMS; therefore, the increase in the number of layers is beneficial for improving the overall compressive strength of the lightweight concrete. In Figure 6b, it can be seen that as the HC-R-EMS wall thickness increases, the density of the concrete composite material also increases. This is because the density of the heavy calcium carbonate-reinforced epoxy resin-curing system outside the EPS is greater than the EPS, so the density of the multi-phase lightweight concrete also increases with the increase in the wall thickness of the HC-R-EMS filled in the multi-phase lightweight concrete. In addition, changes in the density and compressive strength can be seen; the increase in the compressive strength is significantly higher than the change in the density, which means that increasing the wall thickness of the HC-R-EMS without significant changes in the density can make the concrete composite material obtain greater compressive strength.

### 3.5. The Effect of HGMS with Different Volume Ratios on the Compressive Strength of Multi-Phase Lightweight Concrete

**Figure 7:**
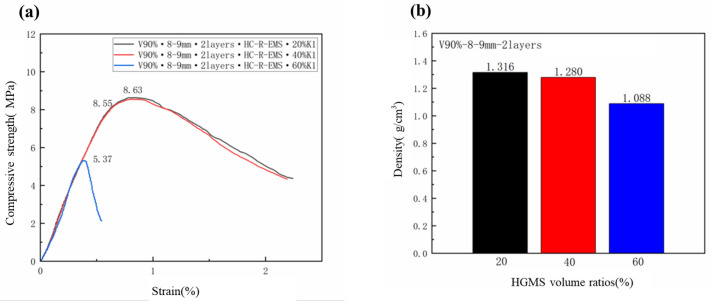
Compressive strength (**a**) and density (**b**) of lightweight concrete filled with different HGMS volume ratios.

In addition to the accumulation volume fraction, thickness. and inner diameter of the HC-R-EMS filled in concrete, the composition of the lightweight concrete matrix also affects the performance of the multi-phase composite lightweight concrete. In this group of experiments, an 8–9 mm diameter enhancement of two layers of the HC-R-EMS with a filling volume fraction of 90% was used. The matrix volume ratios of 20%, 40%, and 60% of K1 grade HGMS and cement mixed were selected as the matrix of the multi-phase composite lightweight concrete. Figure 7a shows the compressive strength of the multi-phase composite lightweight concrete with different HGMS ratios, which are 8.63 MPa (20% HGMS), 8.55 MPa (40% HGMS), and 5.37 MPa (60% HGMS), respectively. Figure 7b shows the density of the multi-phase composite lightweight concrete with different HGMS ratios, which are 1.316 g/cm^3^ (20% HGMS), 1.280 g/cm^3^ (40% HGMS), and 1.088 g/cm^3^ (60% HGMS), respectively. The compressive strength of the lightweight concrete filled with HC-R-EMS decreases with the increase in the HGMS ratio in the matrix. When the filling HGMS ratio is 20%, the strength of the lightweight concrete is the highest, which can reach 8.63 MPa; the compressive strength of the lightweight concrete filled with 40% and 60% HGMS are 99.1% (40%) and 62.2% (60%) of that filled with 20% HGMS, respectively. This is because HGMS is a kind of micro, hollow spherical powder with self-lubricating and non-water-absorbing characteristics. Due to the smooth surface of the HGMS and the insufficient bonding with the cement paste, the concrete with a large amount of HGMS material usually has low strength and brittle failure.

### 3.6. The Effect of Basalt Fiber Length on the Compressive Strength of Multi-Phase Composite Lightweight Concrete

In order to study the effect of the different lengths of basalt fibers on the compressive strength of the multi-phase composite lightweight concrete, this group of experiments used a 90% volume fraction of HC-R-EMS, where HC-R-EMS was reinforced with two layers of heavy calcium carbonate using 8–9 mm EPS, and the matrix was made of a mixture of 40% K1 and 60% cement. Then, 3 mm, 6 mm, 9 mm, and 12 mm basalt fibers were added to the matrix, with a mass fraction of 2.0%. Due to the reduction in the lightness and the ease of the concrete after fiber addition, the water-to-cement ratio was increased in the fiber-added lightweight concrete groups to facilitate the experiment.

**Figure 8:**
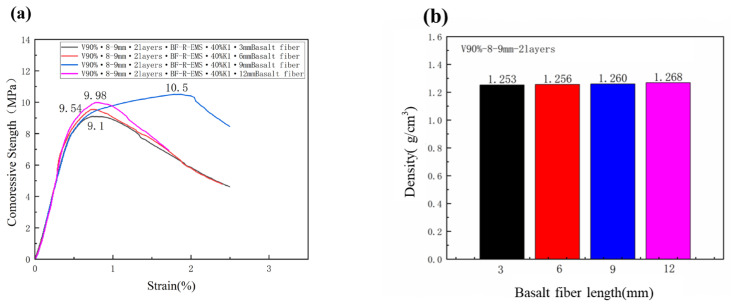
Compressive strength (**a**) and density (**b**) of lightweight concrete filled with different Basalt Fiber Length.

Figure 8a shows the compressive strength of the multi-phase composite lightweight concrete made with different lengths of basalt fibers under the same volume fraction of HC-R-EMS, which were 9.1 MPa (3 mm), 9.54 MPa (6 mm), 10.5 MPa (9 mm), and 9.98 MPa (12 mm), respectively. It can be seen that when the length of the basalt fibers is 3 mm, 6 mm, and 9 mm, the compressive strength of the multi-phase composite lightweight concrete increases with the increase in the fiber length. When the added basalt fiber length is 12 mm, the compressive strength is slightly reduced; this is because the fibers have a crack-blocking effect and the basalt fibers in the HC-R-EMS matrix have a three-dimensional distribution, effectively reducing the stress concentration of the micro-cracks. The basalt fibers are distributed in all regions of the lightweight concrete samples, which together form the whole.

Figure 8b shows the densities of the multi-phase composite lightweight concrete with 3 mm, 6 mm, 9 mm, and 12 mm basalt fibers, which were 1.253 g/cm^3^, 1.256 g/cm^3^, 1.260 g/cm^3^, and 1.268 g/cm^3^, respectively. From the densities of the lightweight concrete blocks with four different lengths of basalt fibers, it can be seen that the longer the length of the added basalt fibers, the denser the lightweight concrete is, but the density change is not obvious.

### 3.7. Effect of Basalt Fiber Content on the Compressive Properties of Multi-Phase Composite Lightweight Concrete

In order to study the effect of the different contents of BF on the compressive strength of the multi-phase composite lightweight concrete, this group of experiments used a 90% volume fraction of HC-R-EMS, in which HC-R-EMS was obtained by enhancing two layers of heavy calcium carbonate with 8–9 mm EPS. The matrix was mixed with HGMS and cement in a volume ratio of 2:3. Then, 0.5%, 1%, 1.5%, and 2% of 9 mm basalt fibers were added to the matrix, respectively. Due to the reduction in the lightweight concrete and plasticity after adding the fibers, this group of experiments increased the water-cement ratio during the preparation of the lightweight concrete to facilitate the experiment.

**Figure 9:**
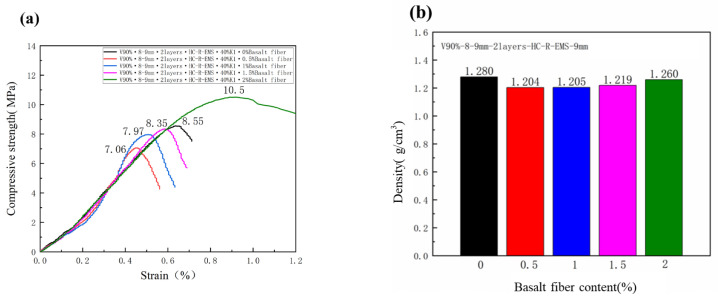
Compressive strength (**a**) and density (**b**) of lightweight concrete filled with different Basalt Fiber content.

Figure 9 shows the density and compressive strength of the lightweight concrete filled with different mass fractions of BF. As shown in the figure, when the content of BF in the matrix is 0, 0.5%, 1.0%, 1.5%, and 2%, the compressive strength and density of the multi-phase composite lightweight concrete are 8.55 MPa, 7.06 Mpa, 7.97 Mpa, 8.35 Mpa, and 10.5 Mpa; 1.280 g/cm^3^, 1.204 g/cm^3^, 1.205 g/cm^3^, 1.219 g/cm^3^, and 1.260 g/cm^3^, respectively.

The density of the test group with BF was reduced due to the increase in the water to ash ratio and was lower than that of the lightweight concrete blocks without BF. Only the lightweight concrete blocks with a 2% mass fraction of BF had a higher compressive strength than the lightweight concrete blocks without the addition of BF.

As the fiber content added to the lightweight concrete specimen increases, the density of the specimen increases. When the BF content is increased from 0.5% to 1% and from 1% to 1.5%, the density does not increase by more than 1%. Increasing the BF content from 1.5% to 2% increases the density by more than 3%. The addition of small amounts of BF does not increase the density of the lightweight concrete specimens too much, but when the BF content is increased, the increase in the density begins to become greater. This is because when the fibers content is increased, there are more fibers on the surface of the lightweight concrete specimen, the pore channels for water from the interior to the surface of the lightweight concrete are blocked, and the water loss and void ratio of the specimen are reduced, increasing the compressive strength of the specimen and the density at the same time.

The greater the content of fibers in the specimen, the more the compressive strength of the lightweight concrete specimen increases and the greater the compressive strength of the specimen. When a light weight concrete specimen is added with a mass fraction of 2% BF, the compressive strength is greatest at this point. This is because when the number of fibers increases, the adhesion within the matrix increases, the matrix is tighter and the strength of the lightweight concrete specimen increases. The increase in the compressive strength of the lightweight concrete specimens was relatively most pronounced when the mass fraction of BF was 2.0%, with a 22.8% increase in the compressive strength compared to the lightweight concrete specimens without BF in the HGMS system. This is due to the ability of the fibers to fill the voids between the concrete, making it more compact.

### 3.8. Compression Resistance Mechanism of HC-R-EMS Filled Multiphase Composite Lightweight Concrete

**Figure 10:**
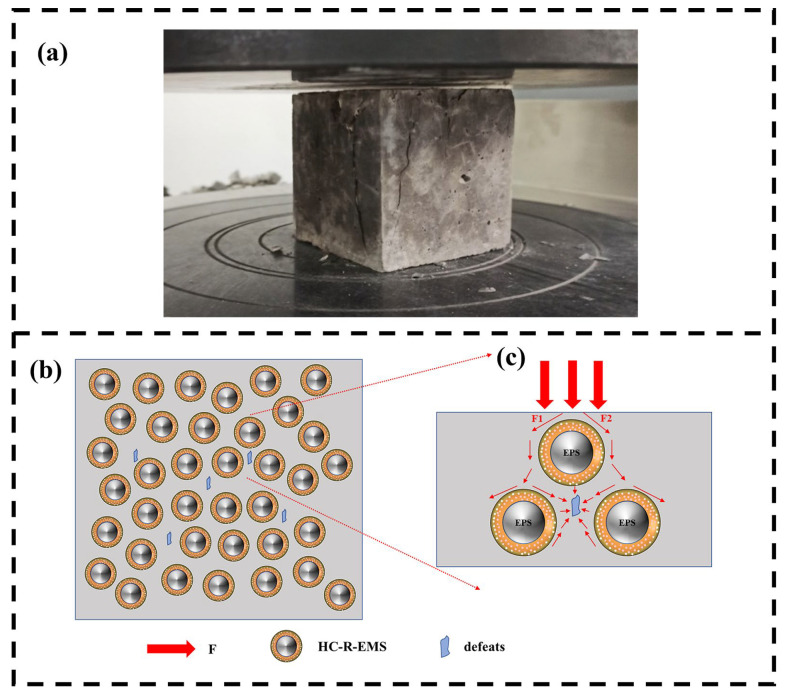
The experimental diagram (**a**) and the compressive mechanism (**b**,**c**) of the HC-R-EMS multiphase composite lightweight concrete.

Figure 10 shows the experimental diagram (Figure 10a) and the compressive mechanism (Figure 10b,c) of the HC-R-EMS multiphase composite lightweight concrete.

Due to the action of EPS and the “ball rolling” preparation process, the inner and outer surfaces of the reinforced epoxy composite spheres are well curved, and when the epoxy composite spheres are compressed by an external force, the external force is transmitted along the surface of the spheres. As shown in Figure 10c, when the concrete is damaged by external forces, the force is rapidly transferred from the concrete matrix to the HC-R-EMS, and then from the HC-R-EMS to the matrix or the next AR-EMS. In addition, there are pore defects in the HGMS-cement matrix, and the force will cause stress concentration at the defects as it is transferred downwards, thus making the defects increasingly larger. Once the cohesive force exceeds the maximum limit of the matrix concrete, the composite will break and eventually expand outwards, causing damage to the whole material; another cause of damage is that the epoxy composite ball is prepared from EPS and reinforcing materials by the “ball rolling method”, the combination itself has a poor resistance to compression. This will further reduce the internal compressive properties of the material.

The purpose of adding fibers is to connect the matrix into a network, thereby increasing the maximum confining force of the matrix concrete. In this paper, basalt fibers were investigated, and the results show that the addition of basalt fibers does have a reinforcing effect on the compressive strength of lightweight concrete. This is mainly due to the basalt fibers having the effect of connecting adjacent epoxy composite balls. Damage to plain concrete blocks under external forces is sudden, brittle, and completely destructive, whereas the addition of basalt fibers inhibits the production of such sudden damage.

### 3.9. HC-R-EMS Filled Composite Lightweight Concrete Cross-Section and Cross-Section Scanning Electron Microscopy

**Figure 11:**
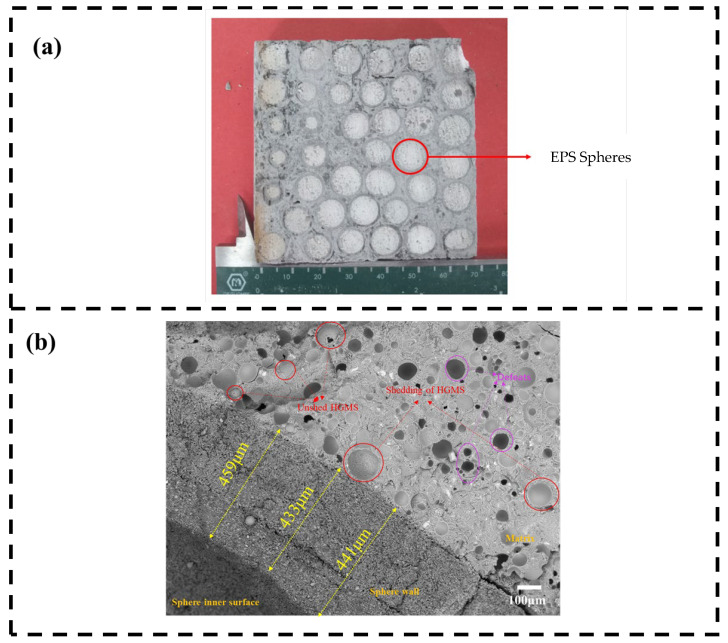
Section (**a**), cross-sectional SEM (**b**) of lightweight concrete.

Figure 11 shows the cross-sectional and cross-sectional SEM images of the HC-R-EMS-filled multiphase composite lightweight concrete. As can be seen in Figure 11a, the HC-R-EMS is uniformly distributed in the matrix and the homogeneous distribution facilitates the overall stability of the structure, which is largely free from bulging due to the limitations of the concrete matrix. It can also be seen that there are a relatively small number of defects and holes present in the matrix, which are due to failures in the manual filling process.

The microstructure in Figure 11b shows that the average wall thickness of the two layers of HC-R-EMS can reach 450 μm. Due to the insolubility of the filled spheres and the cement matrix material, there is a clear demarcation line between the sphere walls and the cement matrix, but the bond between them is still very tight, which is very favorable for the transfer of the forces they are subjected to, giving them good compressive resistance. Multiple fractures occurred due to the brittle fracture, some HGMS dispersed in the matrix remained intact and the fractured aerogel stayed in the matrix, forming a groove. In addition, the presence of a few defects does not affect the compressive resistance.

## 4. Conclusions

In this paper, HC-R-EMS was prepared by the ball rolling method and filled into the matrix of the HGMS-cement system as an aggregate, and then basalt fibers were added to prepare a multiphase composite concrete material. The relationship between the stacking volume fraction of the HC-R-EMS, the initial internal diameter of the epoxy composite ball, the number of reinforcing layers of HC-R-EMS, the volume ratio of the HGMS, the length and content of basalt fibers, and the compressive properties of the composite lightweight concrete were investigated. The main findings are summarized below.

(1) The strength and density of the lightweight concrete specimens prepared by filling with HC-R-EMS are related to the volume fraction of the stacking of the HC-R-EMS, the initial inner diameter of the epoxy composite spheres, and the number of layers of reinforcement of the HC-R-EMS. The compressive strength and density of the lightweight concrete specimens were also reduced. The density and maximum compressive strength of the lightweight concrete at a 90% volume fraction of HC-R-EMS, 8–9 mm initial internal diameter, and three layers of HC-R-EMS reinforcement were 0.953 g/cm^3^ and 12.67 MPa, respectively, which were 57% lower in density and 73% lower in compressive strength compared to the concrete with 0 volume fraction of HC-R-EMS. Although the compressive strength was reduced, the concrete had the lowest density and the highest compressive strength compared to the other experimental groups under these conditions.
(2) As the volume proportion of the HGMS increases, the density and compressive strength of the multi-phase composite concrete decreases, and when the volume fraction of the HGMS is 60%, the overall density and compressive strength of the concrete decreases significantly due to the smooth surface of the HGMS and the lack of bonding with the cement paste.
(3) The addition of basalt short-cut fibers to the concrete matrix can improve the strength of lightweight concrete specimens. With the increase in the fiber length and mass fraction, the strength of the lightweight concrete specimens will increase, but the addition of fibers will also reduce the ease of the concrete, increasing the difficulty of production and the need to increase the water-cement ratio at the same time. The higher the fiber content, the higher the density and strength; the longer the fiber length, the higher the density of the lightweight concrete specimens, but the increase is not significant, with the maximum strength occurring at 9 mm fiber length.

In this work, the effect of an increase in fiber on the strength of lightweight concrete was investigated within a certain range, and the compressive properties of lightweight concrete became stronger with the increase in the basalt fiber content, but the threshold value of the fiber content was not explored, and further tests can be conducted in the future to investigate to what extent the increase in the fiber content will seriously affect the compressive strength of concrete. In addition, tensile strength and bending strength are also important indicators of concrete, which can be considered together in the future to evaluate the overall performance of concrete.

## Figures and Tables

**Table 1:** Multiphase composite lightweight concrete specimens with different parameters.

Sample	HGMS in Filler (wt%)	HC-R-EMS (svol%)	Layers	Diameter (mm)	BF’s Length (mm)	BF (wt%)
1	K1-40	0	2	8–9	-	-
2	K1-40	20	2	8–9	-	-
3	K1-40	40	2	8–9	-	-
4	K1-40	60	2	8–9	-	-
5	K1-40	80	2	8–9	-	-
6	K1-40	90	2	8–9	-	-
7	K1-40	90	2	10–11	-	-
8	K1-40	90	0	8–9	-	-
9	K1-40	90	1	8–9	-	-
10	K1-40	90	3	8–9	-	-
11	K1-20	90	2	8–9	-	-
12	K1-60	90	2	8–9	-	-
13	K1-40	90	2	8–9	3	2.0
14	K1-40	90	2	8–9	6	2.0
15	K1-40	90	2	8–9	9	2.0
16	K1-40	90	2	8–9	9	2.0
17	K1-40	90	2	8–9	9	0
18	K1-40	90	2	8–9	9	0.5
19	K1-40	90	2	8–9	9	1.0
20	K1-40	90	2	8–9	9	1.5
21	K1-40	90	2	8–9	9	2

## Data Availability

The data presented in this study are available on request from the corresponding author.
